# Evaluation of the CNS-LAND Score for ICU Mortality Prediction in Patients with Spontaneous Intracerebral Hemorrhage

**DOI:** 10.3390/jcm15145690

**Published:** 2026-07-20

**Authors:** Mehtap Zengi, Gülbahar Çalışkan

**Affiliations:** Intensive Care Unit, Department of Anesthesiology and Reanimation, Bursa City Hospital, Doğanköy İç Yolu, Nilüfer, Bursa 16110, Türkiye; alkanbahar@yahoo.com

**Keywords:** intracerebral hemorrhage, intensive care unit mortality, CNS-LAND score, prognostic scoring, ROC analysis

## Abstract

**Objectives**: This study investigated the prognostic value of the CNS-LAND score in predicting Intensive Care Unit (ICU) mortality among patients admitted with spontaneous intracerebral hemorrhage (ICH). **Methods**: In this single-center, retrospective cohort study, 305 adult ICU patients with spontaneous ICH (September 2022–March 2026) were included. The primary endpoint was ICU mortality. Prognostic performance was evaluated using receiver operating characteristic (ROC) curve analysis and the bootstrap-based DeLong method. The independent contribution of CNS-LAND beyond the Glasgow Coma Scale (GCS) was assessed using partial correlation and logistic regression analyses. **Results**: ICU mortality occurred in 71 of 305 patients (23.3%; median age 66.0 years). CNS-LAND scores were significantly higher in non-survivors than survivors (median 6.0 vs. 3.0, *p* < 0.001). ROC analysis showed the highest discriminative performance for GCS (AUC: 0.925), followed by Sequential Organ Failure Assessment (SOFA) (AUC: 0.906), Acute Physiology and Chronic Health Evaluation II (APACHE II) (AUC: 0.840), and CNS-LAND (AUC: 0.816; *p* = 0.467 vs. APACHE II). At a cutoff of ≥5, CNS-LAND yielded 83.1% sensitivity, 70.9% specificity, and a negative predictive value of 93.3%. Spearman analysis revealed near-perfect collinearity between National Institutes of Health Stroke Scale (NIHSS) and GCS (rs = −0.964, *p* < 0.001). After controlling for GCS, CNS-LAND lost statistical significance (partial rs = −0.051, *p* = 0.373), and logistic regression confirmed no independent prognostic contribution beyond GCS (LRT: χ^2^ = 0.121, *p* = 0.728). **Conclusions**: CNS-LAND demonstrated mortality prediction performance comparable to APACHE II in critically ill ICH patients. However, this apparent performance was almost entirely attributable to the score’s NIHSS component: NIHSS and GCS were near-perfectly collinear (rs = −0.964), and after adjustment for GCS, CNS-LAND was no longer significantly associated with mortality (partial rs = −0.051, *p* = 0.373; likelihood-ratio test *p* = 0.728; ΔAUC ≈ 0.000). CNS-LAND therefore provides essentially no independent prognostic information beyond GCS in this population, indicating that its discriminative performance is redundant with, rather than complementary to, standard neurological assessment. This redundancy is rooted in the parallel progression of neurological deficit and consciousness characteristic of severe ICH—a relationship fundamentally distinct from the acute ischemic stroke population in which CNS-LAND was originally derived.

## 1. Introduction

Stroke remains the leading cause of death and permanent disability among neurological disorders worldwide, and early diagnosis, rapid treatment, and accurate prognostic assessment constitute the cornerstones of contemporary stroke management. Although spontaneous intracerebral hemorrhage (ICH) accounts for only approximately 10–15% of all stroke cases, it is associated with substantially higher mortality rates and more severe functional impairment than other stroke subtypes [1]. The 30-day mortality rate of ICH has been reported as approximately 40%, with considerable variation across populations [2]. Among patients requiring admission to the intensive care unit (ICU), the clinical burden becomes even more critical, with early mortality rates exceeding 30%. Consequently, the search for reliable and practical tools for mortality prediction in this patient population remains an important clinical priority.

Several prognostic scoring systems integrating clinical and radiological parameters have been developed to predict outcomes in patients with ICH. Among the most widely used tools in clinical practice are the National Institutes of Health Stroke Scale (NIHSS) and the Glasgow Coma Scale (GCS), both of which provide objective assessments of neurological status at presentation and serve as important predictors of short- and long-term outcomes [3,4]. In critical care settings, general ICU scoring systems such as the Sequential Organ Failure Assessment (SOFA) score and the Acute Physiology and Chronic Health Evaluation (APACHE) II score are commonly employed [5,6]. However, calculation of these scores requires the simultaneous evaluation of multiple physiological and laboratory variables, which may limit their practical applicability, particularly in acute care settings. Moreover, SOFA and APACHE II were originally developed for heterogeneous ICU populations and were not specifically designed to capture the pathophysiological processes unique to ICH, including neuroinflammation, coagulation abnormalities, and remote organ injury. This limitation may inherently restrict their prognostic utility in patients with ICH. Therefore, there remains a need for novel prognostic tools that can comprehensively reflect the pathophysiology of ICH while maintaining simplicity and clinical practicality.

Recent studies have demonstrated that, in addition to clinical assessment scales, biochemical biomarkers may provide valuable prognostic information in stroke. Stroke is increasingly recognized not merely as an isolated neurological event but as a systemic disease process characterized by neuronal injury, neuroinflammation, and oxidative stress, accompanied by peripheral immune dysregulation, autonomic nervous system dysfunction, and distant organ damage. Within this framework, biomarkers reflecting remote organ injury, such as lactate dehydrogenase (LDH), creatine kinase-MB (CK-MB), and alanine aminotransferase (ALT), as well as markers of inflammation and coagulation, including neutrophil percentage, D-dimer, C-reactive protein (CRP), and procalcitonin, have been reported to be significantly associated with clinical outcomes and mortality in patients with ICH [7,8].

Based on these findings, integrated prognostic models combining clinical assessment parameters with multisystem biomarkers have been developed and evaluated. One such model is the CNS-LAND score, a 9-point composite scoring system incorporating CK-MB, NIHSS score, systolic blood pressure, LDH, ALT, neutrophil percentage, and D-dimer levels. The score was originally designed to predict early neurological deterioration (END) following intravenous thrombolytic therapy (IVT) in patients with acute ischemic stroke and demonstrated strong discriminative performance in both derivation and evaluation cohorts [9]. By simultaneously assessing neurological status and systemic biological responses, the CNS-LAND score represents a practical prognostic tool that relies solely on routinely available clinical and laboratory data, without requiring advanced diagnostic infrastructure.

Nevertheless, the validity of the CNS-LAND score in patients with ICH, particularly within critically ill ICU populations, has not yet been investigated. Given that systemic inflammation, organ injury, and coagulation disturbances are often more pronounced in ICH than in ischemic stroke, it may be hypothesized that the CNS-LAND score could also capture key pathophysiological features of ICH. Indeed, previous studies have demonstrated the independent prognostic significance of several CNS-LAND components—including LDH, CK-MB, and D-dimer—in patients with ICH [7,8,10]. However, the contribution of these variables within a unified scoring framework for predicting mortality in ICH has not been systematically evaluated.

Accordingly, in the present study, we investigated whether the CNS-LAND score, originally developed for acute ischemic stroke, could predict ICU mortality in patients with spontaneous ICH, a distinct clinical entity, particularly among severe cases requiring intensive care management. To this end, we compared the prognostic performance of the CNS-LAND score with that of the GCS, which directly reflects neurological severity; the SOFA score, which assesses multiorgan dysfunction; and the APACHE II score, a widely used predictor of ICU mortality.

## 2. Materials and Methods

### 2.1. Study Design and Ethical Approval

This study was designed as a single-center, retrospective observational cohort study. The study protocol was approved by the local Ethics Committee (Decision No: 2026-06/1), and all procedures were conducted in accordance with the principles of the Declaration of Helsinki. Patient data were obtained from the institution’s electronic health record system and medical files. Due to the retrospective nature of the study, the requirement for individual informed consent was waived by the Ethics Committee.

### 2.2. Patient Selection and Study Population

All consecutive adult patients admitted to the intensive care unit (ICU) of our hospital with a diagnosis of spontaneous intracerebral hemorrhage (ICH) between 1 September 2022 and 1 March 2026 were screened for eligibility. The diagnosis of ICH was confirmed through the combined assessment of clinical findings at presentation and neuroimaging results obtained by brain computed tomography (CT) and/or magnetic resonance imaging (MRI).

Inclusion criteria: Patients were eligible for inclusion if they were aged 18 years or older, had neuroimaging-confirmed supratentorial spontaneous (non-traumatic) intracerebral hemorrhage, required admission to the intensive care unit (ICU), and had complete routine biochemical and hematological laboratory data available at the time of admission.

Exclusion criteria: Patients were excluded if they had traumatic intracerebral hemorrhage, ischemic stroke with hemorrhagic transformation, secondary intracerebral hemorrhage caused by a brain tumor, arteriovenous malformation (AVM), or cavernoma, incomplete clinical or laboratory data, or death within the first 24 h after admission.

### 2.3. Collection of Demographic and Clinical Data

Demographic data, including age and sex, were recorded for all patients. Information regarding concomitant systemic diseases, including hypertension, diabetes mellitus, atrial fibrillation, coronary artery disease, and chronic kidney disease, was retrospectively obtained from medical records. Comorbidity burden was categorized into three groups: no comorbidity, a single comorbidity, and multiple comorbidities (≥2).

Neurological assessment included the Glasgow Coma Scale (GCS) score, National Institutes of Health Stroke Scale (NIHSS) score, and systolic blood pressure (mmHg) at admission, all of which were recorded simultaneously. Intensive care unit (ICU) follow-up parameters, including total ICU length of stay (days) and the requirement for mechanical ventilation (MV status was recorded at ICU admission), were also documented for each patient.

### 2.4. Laboratory Parameters

For all analyses, the worst laboratory values recorded within the first 24 h following ICU admission were used. Hematological parameters included white blood cell count (WBC, ×10^9^/L), (%) platelet count (×10^9^/L). Biochemical parameters included blood urea nitrogen (BUN), creatinine, aspartate aminotransferase (AST), alanine aminotransferase (ALT), C-reactive protein (CRP), procalcitonin, lactate dehydrogenase (LDH) and creatine kinase-MB isoenzyme (CK-MB, ng/mL). Coagulation parameters included prothrombin time (PT), international normalized ratio (INR), activated partial thromboplastin time (aPTT), and D-dimer (mg/L).

### 2.5. Calculation of Prognostic Scores

The CNS-LAND, APACHE II, and SOFA scores were calculated for each patient using the worst clinical and laboratory data obtained during the first 24 h after admission. The Glasgow Coma Scale (GCS) was included as a fourth comparator because it is a component of both the APACHE II and SOFA scoring systems and is also widely recognized as an independent indicator of neurological severity.

The CNS-LAND score was calculated as a 9-point scale based on seven independent variables, as originally described by Jin et al. [9]. The cutoff values and corresponding point assignments were as follows: CK-MB ≥ 5 ng/mL (+1 point), NIHSS score 5–15 (+1 point) or 16–42 (+3 points), systolic blood pressure ≥ 160 mmHg (+1 point), LDH ≥ 220 U/L (+1 point), ALT ≥ 40 U/L (+1 point), neutrophil percentage ≥ 80% (+1 point), and D-dimer ≥ 0.5 mg/L (+1 point). Total scores were categorized as low risk (0–3 points) and high risk (4–9 points).

It should be noted that the CNS-LAND score was originally developed to predict early neurological deterioration (END) in patients with acute ischemic stroke treated with intravenous thrombolytic therapy (IVT). In the present study, however, the score was applied to a different patient population (spontaneous ICH requiring ICU admission) and evaluated against a different clinical endpoint (ICU mortality).

### 2.6. Primary and Secondary Endpoints

The primary endpoint was ICU mortality.

The secondary endpoints included total ICU length of stay (days) and the requirement for mechanical ventilation, both of which were compared between survivors and non-survivors. In addition, the prognostic performance of the CNS-LAND, GCS, APACHE II, and SOFA scores in predicting ICU mortality was evaluated as a secondary outcome.

### 2.7. Statistical Analysis

The normality of continuous variables was assessed using the Kolmogorov–Smirnov test. All continuous variables failed to meet the assumption of normality; therefore, they are presented as median (interquartile range [IQR]: Q1–Q3). Categorical variables are expressed as number and percentage (*n*, %). Between-group comparisons of continuous variables (survivors vs. non-survivors) were performed using the Mann–Whitney U test, while categorical variables were compared using the chi-square test or Fisher’s exact test, as appropriate.

The discriminative performance of each prognostic score (CNS-LAND, GCS, SOFA, and APACHE II) for predicting ICU mortality was evaluated by receiver operating characteristic (ROC) curve analysis. The area under the ROC curve (AUC) with 95% confidence intervals (CI) was calculated using the nonparametric method. The optimal cut-off value for each score was determined by maximizing the Youden J index (J = sensitivity + specificity − 1). Sensitivity, specificity, positive predictive value (PPV), and negative predictive value (NPV) were reported at the optimal cut-off. ROC curves were generated using Python (version 3.12.3) with the following libraries: SciPy (version 1.17.1) and Matplotlib (version 3.10.8). Pairwise comparisons of AUC values between CNS-LAND and each comparator score were performed using the bootstrap-based DeLong method (2000 iterations) in MedCalc Statistical Software. Positive and negative likelihood ratios (PLR and NLR) were calculated at the optimal cut-off for each score, as these measures of diagnostic accuracy are independent of disease prevalence, unlike PPV and NPV.

Associations between prognostic scores, and between CNS-LAND and ICU mortality, were assessed using Spearman’s rank correlation coefficient (rs). To determine whether CNS-LAND provided discriminatory information independent of GCS, partial Spearman correlation analysis was performed with GCS as the controlling variable, using a two-step rank-transformation approach. The incremental contribution of CNS-LAND beyond GCS was further quantified using the Integrated Discrimination Improvement (IDI).

To evaluate whether CNS-LAND provided independent prognostic information beyond established clinical predictors, two complementary logistic regression analyses were performed. In the primary analysis, a GCS-only model (Model 1) was compared with a model additionally incorporating CNS-LAND (Model 2). In the extended analysis, a broader multivariable base model incorporating GCS, age, and comorbidity burden (Model A) was compared with a model additionally including CNS-LAND (Model B). Mechanical ventilation was excluded from all regression models due to complete separation—all non-survivors required mechanical ventilation, precluding parameter estimation. SOFA and APACHE II were likewise excluded as covariates because both scores incorporate GCS directly, which would introduce collinearity with the primary predictor of interest. Incremental model performance was assessed using the likelihood ratio test (LRT).

To assess model calibration, the Hosmer-Lemeshow goodness-of-fit test (10 groups, df = 8) was applied to each prognostic score, and calibration plots depicting observed versus predicted ICU mortality across deciles of predicted probability were constructed. The Brier score was calculated as a composite measure of calibration and discrimination.

Decision curve analysis (DCA) was performed to evaluate the net clinical benefit of each prognostic score across a range of threshold probabilities (5–80%), relative to the ‘treat-all’ and ‘treat-none’ strategies.

All analyses were performed using IBM SPSS Statistics version 26.0 (IBM Corp., Armonk, NY, USA) and MedCalc Statistical Software version 23.5.5 (MedCalc Software bv, Ostend, Belgium). A two-tailed *p* value < 0.05 was considered statistically significant.

## 3. Results

### 3.1. Patient Characteristics

**Figure 1:**
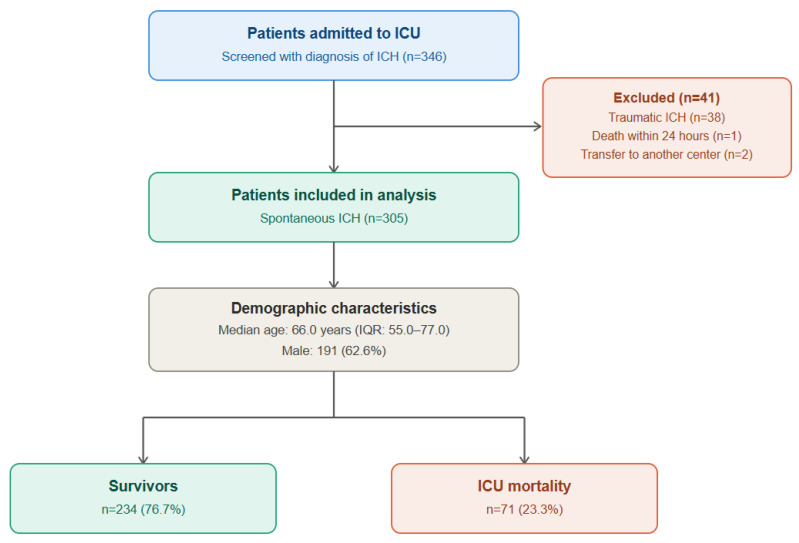
Patient selection flowchart. ICH: intracerebral hemorrhage; ICU: intensive care unit.

A total of 346 patients admitted to the ICU with a diagnosis of intracerebral hemorrhage were screened for eligibility. Of these, 41 were excluded: 38 had traumatic intracerebral hemorrhage, 1 died within the first 24 h of admission, and 2 were transferred to external centers. The remaining 305 patients with non-traumatic spontaneous ICH were included in the final analysis. The median age was 66.0 years (IQR: 55.0–77.0), and 191 patients (62.6%) were male. The large majority of patients (82.6%) had at least one comorbidity, with 161 (52.8%) presenting with multiple comorbidities. ICU mortality occurred in 71 patients (23.3%), while 234 (76.7%) survived to discharge (Figure 1).

Non-survivors had a significantly higher burden of multiple comorbidities (69.0% vs. 47.9%), markedly higher NIHSS scores, and lower admission systolic blood pressure (all *p* < 0.001). Age did not differ significantly between groups (median 68.0 vs. 65.5 years; *p* = 0.067).

The CNS-LAND score was significantly higher in non-survivors compared with survivors (median 6.0 vs. 3.0; *p* < 0.001). Similarly, APACHE II—a validated ICU mortality predictor—was markedly elevated in non-survivors (median 18.0 vs. 10.0; *p* < 0.001). GCS was significantly lower in the non-survivor group (median 4.0 vs. 14.0; *p* < 0.001), consistent with the severity of neurological injury at admission.

All non-survivors (100%) required mechanical ventilation compared with 20.9% of survivors. Among laboratory parameters, LDH, CK-MB, CRP, procalcitonin, BUN, creatinine, INR, PT, and D-dimer were significantly elevated in non-survivors (all *p* ≤ 0.010). Detailed baseline characteristics and between-group comparisons are presented in Table 1.

### 3.2. Discriminative Performance of Prognostic Scores

**Figure 2:**
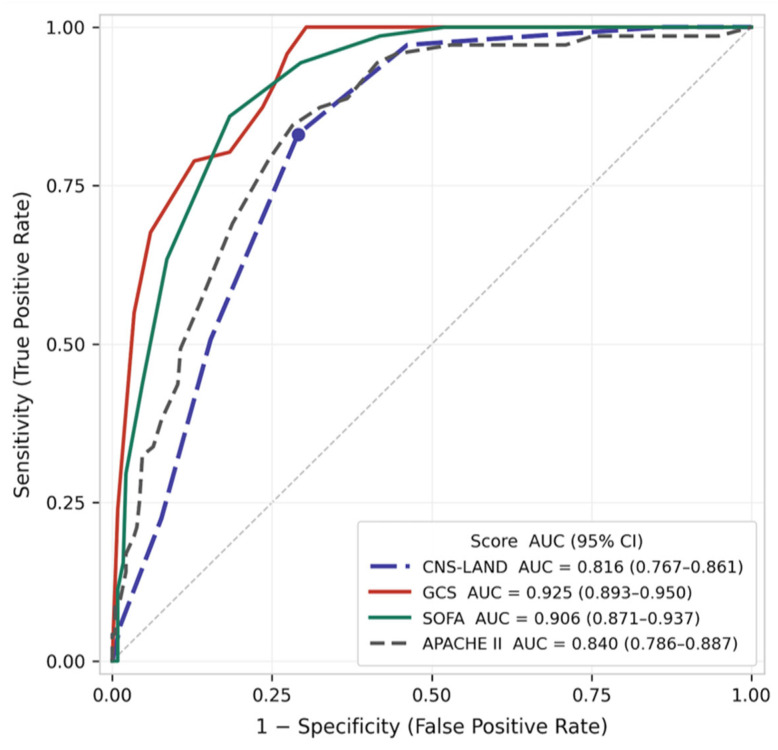
Receiver operating characteristic (ROC) curves for ICU mortality prediction in ICU patients with non-traumatic spontaneous intracerebral hemorrhage (*n* = 305). Curves are shown for GCS, SOFA, APACHE II, and CNS-LAND. The filled circle on the CNS-LAND curve indicates the optimal cut-off point (score ≥ 5) determined by the Youden J index. AUC values are presented with 95% confidence intervals derived from 2000-iteration bootstrap resampling. The dashed diagonal represents chance-level discrimination.

All four prognostic scores—GCS, SOFA, APACHE II, and CNS-LAND—differed significantly between survivors and non-survivors (all *p* < 0.001, Table 1). ROC analysis demonstrated that GCS achieved the highest discriminative performance for ICU mortality (AUC: 0.925; 95% CI: 0.893–0.950), followed by SOFA (AUC: 0.906; 95% CI: 0.871–0.937), APACHE II (AUC: 0.840; 95% CI: 0.786–0.887), and CNS-LAND (AUC: 0.816; 95% CI: 0.767–0.861). At an optimal cut-off of ≥5, the CNS-LAND score yielded a sensitivity of 83.1%, specificity of 70.9%, and a negative predictive value of 93.3%, indicating that a score below this threshold reliably identified patients at low risk of ICU mortality. Full ROC performance metrics are detailed in Table 2 and illustrated in Figure 2.

Pairwise AUC comparisons using the bootstrap-based DeLong method revealed that GCS and SOFA each significantly outperformed CNS-LAND (ΔAUC: −0.109 [95% CI: −0.151 to −0.067], *p* < 0.001; and ΔAUC: −0.090 [−0.138 to −0.040], *p* < 0.001, respectively). In contrast, the discriminative performance of CNS-LAND was not significantly different from that of APACHE II (ΔAUC: −0.024 [95% CI: −0.088 to +0.040]; *p* = 0.467). Pairwise comparisons are summarized in Table 3.

### 3.3. Correlation Analysis and Independent Contribution of CNS-LAND

CNS-LAND showed significant correlations with all comparator severity scores: GCS (rs = −0.772), SOFA (rs = +0.666), and APACHE II (rs = +0.577; all *p* < 0.001). Notably, NIHSS—the dominant component of the CNS-LAND score—demonstrated near-perfect collinearity with GCS in this cohort (rs = −0.964, *p* < 0.001), suggesting that these two neurological scales carry nearly identical information in patients with space-occupying intracerebral hemorrhage.

Although CNS-LAND was significantly correlated with ICU mortality on univariate analysis (rs = +0.468, *p* < 0.001), this association was no longer statistically significant after controlling for GCS (partial rs = −0.051, *p* = 0.373). In the primary logistic regression analysis, adding CNS-LAND to a GCS-only model did not improve discriminative performance (ΔAUC = 0.000 [95% CI: −0.002 to +0.004]; LRT χ^2^ = 0.121, *p* = 0.728; IDI = 0.001; Table 4).

To address the possibility that this finding was attributable to model underspecification, a broader multivariable model was constructed incorporating GCS, age, and comorbidity burden (Model A: AUC = 0.928). Mechanical ventilation was excluded due to complete separation—all 71 non-survivors (100%) required mechanical ventilation, precluding logistic regression estimation. SOFA and APACHE II were not included as additional covariates because both scores incorporate GCS directly as a sub-component, which would introduce substantial collinearity with the primary predictor of interest. In this extended model, CNS-LAND remained a non-significant predictor (OR = 0.941 [95% CI: 0.701–1.265]; *p* = 0.688), and the likelihood ratio test confirmed no significant improvement in model fit (LRT χ^2^ = 0.161, *p* = 0.688; Table 4). Across both primary and extended regression analyses, GCS was the only statistically significant independent predictor of ICU mortality (OR = 0.583 [95% CI: 0.507–0.669]; *p* < 0.001).

### 3.4. Calibration and Decision Curve Analysis

**Figure 3:**
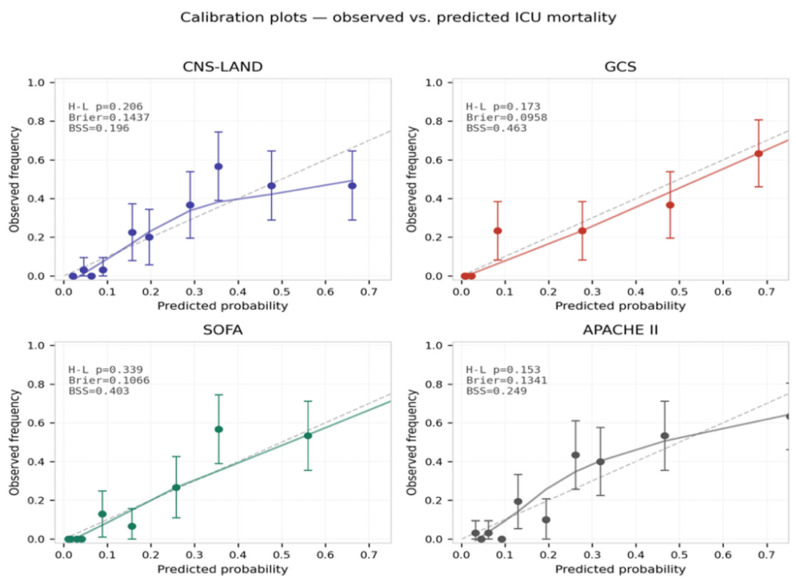
Calibration plots showing observed versus predicted ICU mortality across deciles of predicted probability for each prognostic score (*n* = 305). Points represent observed mortality rates in each decile with 95% confidence intervals; the solid curve represents a LOWESS smooth fit. The dashed diagonal represents perfect calibration. Hosmer-Lemeshow *p* values and Brier scores are displayed within each panel. All four scores demonstrated adequate calibration (all *p* > 0.05).

All four prognostic scores demonstrated adequate calibration, as assessed by both the Hosmer-Lemeshow goodness-of-fit test (all *p* > 0.05; Table 5) and visual inspection of calibration plots (Figure 3). CNS-LAND showed good calibration across deciles of predicted probability (H-L χ^2^ = 10.931; *p* = 0.206), indicating that predicted probabilities corresponded well to observed mortality rates. The Brier score for CNS-LAND was 0.144, compared with 0.096 for GCS, 0.107 for SOFA, and 0.134 for APACHE II. While all scores demonstrated adequate calibration, these Brier scores reflect the overall predictive accuracy of each model and are consistent with the AUC-based discrimination findings: GCS and SOFA demonstrated superior composite predictive performance.

CNS-LAND risk stratification revealed a clinically meaningful gradient of observed ICU mortality across score categories. Among patients with a score of 0–3 (low-risk category), ICU mortality was 1.6% (2/128), whereas among those with a score of 4–9 (high-risk category), mortality was 39.0% (69/177). Within the high-risk group, mortality increased progressively with score: 20.0% at score 4, 41.8% at score 5, 52.6% at score 6, 44.4% at score 7, and 57.1% at score 8, demonstrating an ordinal relationship between CNS-LAND score and ICU mortality risk.

Decision curve analysis demonstrated that all four scores provided net clinical benefit over the treat-all and treat-none strategies across a range of threshold probabilities (Figure 3). At a threshold probability of 20%, CNS-LAND (net benefit: 0.137) provided comparable net benefit to APACHE II (0.142), while GCS (0.158) and SOFA (0.164) showed marginally higher net benefit. However, at threshold probabilities ≥ 30%, the net benefit of CNS-LAND diminished markedly (net benefit at 30%: 0.067; at 50%: −0.007), whereas GCS maintained meaningful net benefit across a substantially wider range of decision thresholds (net benefit at 30%: 0.126; at 50%: 0.111). This pattern supports the utility of CNS-LAND primarily as a rule-out instrument at lower risk thresholds, with GCS demonstrating superior and more consistent clinical net benefit across the full spectrum of decision-relevant threshold probabilities.

## 4. Discussion

The present study investigated whether the CNS-LAND score—originally developed to predict early neurological deterioration following intravenous thrombolysis in acute ischemic stroke—could be extended to forecast ICU mortality in a clinically distinct population of patients with spontaneous intracerebral hemorrhage. Our analyses yielded several important findings. The CNS-LAND score demonstrated clinically meaningful performance in predicting mortality in this patient population, and the difference in AUC compared with APACHE II did not reach statistical significance. Considering their biological proximity to the outcome and their structural characteristics, the superior discriminative performance of the GCS and the SOFA score may be regarded as an expected finding. Perhaps the most important observation of this study, and the one that most directly qualifies CNS-LAND’s apparent performance, was that the association between CNS-LAND and mortality lost statistical significance after adjustment for GCS.

To provide a prevalence-independent assessment of diagnostic accuracy, we additionally calculated the positive and negative likelihood ratios for CNS-LAND (PLR = 2.86; NLR = 0.238). The NLR of 0.238 indicates that a CNS-LAND score below the optimal cut-off produces a moderate, clinically meaningful reduction in the post-test probability of ICU mortality, independent of the underlying event rate. By comparison, GCS demonstrated a markedly lower NLR (0.00) at its optimal threshold, reflecting its perfect sensitivity in this cohort, while SOFA showed the highest PLR (4.68) among the four scores. These likelihood ratios should be applied to the pre-test probability relevant to each clinical setting to derive a more clinically interpretable post-test probability of mortality, rather than relying on NPV alone.

The independent prognostic value of several CNS-LAND components, including LDH, CK-MB, and D-dimer, has previously been demonstrated in patients with ICH [7,8,10]. Unlike ischemic stroke, ICH is characterized by intraparenchymal blood accumulation, the toxic effects of hemoglobin degradation products, and acute disruption of the blood–brain barrier, all of which trigger a profound systemic response that promotes neuroinflammation, coagulopathy, and remote organ injury [11]. This biological framework provides a mechanistic basis for the observed associations. However, not all five components appear to contribute equally. LDH, CK-MB, and D-dimer levels were significantly higher in non-survivors, whereas ALT levels and neutrophil percentage did not differ significantly between groups. This asymmetry suggests that the individual components of the score do not provide equivalent prognostic contributions within the ICH population.

The three CNS-LAND components found to be significant are consistent with the existing literature. The median LDH level in the non-survivor group (296 U/L) markedly exceeded that of the survivor group (214 U/L), aligning with Feng et al.’s study of 406 ICH patients from the MIMIC-IV database, which demonstrated that the highest LDH quartile was associated with a significantly increased risk of 28-day mortality (HR: 3.054; 95% CI: 1.522–6.126; *p* = 0.002) [7]. The higher CK-MB levels observed in the non-survivor group (3.4 vs. 2.1 ng/mL) likely reflect the neurocardiac stress response following cerebral injury, as recent studies have highlighted that brain injury predisposes to catecholamine-mediated myocardial damage [8]. D-dimer levels were found to be approximately twice as high in non-survivors, consistent with Delgado et al.’s prospective study of 98 acute ICH patients, which demonstrated an independent association between elevated D-dimer levels and both early neurological deterioration and first-week mortality [10]. In contrast, the lack of a significant difference in ALT between groups suggests a limited role for this biomarker in ICH prognosis. This observation aligns with Liu et al.’s study of 639 spontaneous ICH patients, which reported that among liver function markers, AST and ALP were associated with clinical outcomes, whereas ALT did not emerge as an independent predictor [12]. Regarding neutrophil percentage, the already elevated baseline neutrophil activation in critically ill ICH patients may have masked intergroup differences. Indeed, the ICH literature suggests that the neutrophil-to-lymphocyte ratio, rather than neutrophil percentage alone, serves as a stronger prognostic indicator; in a meta-analysis encompassing 21 studies and 7176 patients, Guo et al. found that elevated neutrophil-to-lymphocyte was significantly associated with mortality (OR: 1.10; 95% CI: 1.04–1.17) and poor functional outcome (OR: 1.29; 95% CI: 1.17–1.41) [13]. These findings underscore the need to re-evaluate the relative biological weighting of CNS-LAND components in the ICH population in future studies.

Taken together, these findings indicate that the apparent discriminative performance of CNS-LAND in this cohort is not attributable to the score as a composite construct, but is almost entirely driven by a single component: the NIHSS. To state this as plainly as possible: CNS-LAND does not “work” in ICH because its biochemical components (CK-MB, LDH, ALT, neutrophil percentage, D-dimer) meaningfully track mortality; it appears to work only because it inherits, through its NIHSS sub-item, information that GCS already provides. Once that shared information is removed, no independent signal remains. Consequently, when GCS is accounted for, CNS-LAND does not provide independent discriminative information regarding mortality: the partial Spearman correlation with mortality decreased to −0.051 (*p* = 0.373), and binary logistic regression confirmed that adding CNS-LAND to a model containing GCS alone did not significantly improve discriminative performance (ΔAUC = 0.000; LRT χ^2^ = 0.121, *p* = 0.728; IDI = 0.001). This pattern contrasts sharply with the context in which CNS-LAND was originally developed—acute ischemic stroke—where NIHSS can be markedly elevated due to aphasia or hemiplegia while consciousness remains largely intact, allowing for meaningful dissociation between NIHSS and GCS. In ICH, however, the presence of mass effect and progressive herniation leads to parallel progression of neurological deficits and impaired consciousness, rendering GCS and NIHSS functionally overlapping scales. Indeed, previous studies have reported a strong correlation between NIHSS and GCS in ICH patients (r = −0.773; *p* < 0.001) and suggested that this relationship may be even more pronounced in severe ICH cases requiring intensive care [14]. The higher correlation coefficient observed in our study (r = −0.964) can be attributed to our cohort, which predominantly included severe ICU-level cases where deficits in consciousness and focal neurological function progressed concurrently.

Similarly, the superior discriminative performance of the SOFA score (AUC: 0.906) is biologically plausible and consistent with its intended clinical application. In a prospective study of 352 mixed ICU patients, Ferreira et al. demonstrated that SOFA is a robust predictor of mortality across the general critical care population [5]. The present findings extend this observation to patients with spontaneous ICH, further supporting the utility of SOFA as a reliable prognostic tool in neurocritical care settings. It is worth acknowledging that SOFA incorporates GCS as its neurological component, and APACHE II includes neurological assessment indirectly. This overlap may partially explain their superior performance; nonetheless, both scores reflect multiorgan dysfunction beyond neurological status alone, which likely accounts for their incremental discriminative value over CNS-LAND in this critically ill population.

The absence of a statistically significant difference between CNS-LAND and APACHE II (AUC: 0.816 vs. 0.840; *p* = 0.467) represents one of the notable findings of our study. However, this result should not be interpreted as evidence of statistical equivalence. Given the relatively wide confidence interval (95% CI: −0.088 to +0.040), the possibility that APACHE II may be truly superior cannot be excluded. Nevertheless, the lack of a significant performance difference between CNS-LAND—comprising only seven routinely available biochemical parameters—and APACHE II, a comprehensive scoring system incorporating 12 physiological variables in addition to age and chronic health status, may suggest a practical advantage in terms of ease of calculation and clinical applicability [6]. Two principal mechanisms may explain the comparable performance of CNS-LAND and APACHE II. First, the significantly higher levels of inflammatory biomarkers not included in the CNS-LAND framework, namely C-reactive protein (CRP) and procalcitonin, in the non-survivor group—CRP: 12.5 vs. 8.6 mg/L (*p* = 0.013); procalcitonin: 0.2 vs. 0.1 ng/mL (*p* < 0.001)—suggest that the systemic inflammatory response in severe ICH extends beyond the biochemical domains captured by currently available scoring systems. This observation is consistent with the meta-analysis by Guo and Zou involving 25,928 patients, which demonstrated that elevated neutrophil-to-lymphocyte ratio (NLR), white blood cell count (WBC), and CRP levels were associated with poor functional outcomes and increased mortality [15]. Second, the inclusion within CNS-LAND of biomarkers such as LDH, CK-MB, and D-dimer—which directly reflect the systemic response triggered by hemorrhagic stroke—may explain why the broader physiological coverage of APACHE II fails to provide additional discriminative value in this population. This finding is also in agreement with the observations of Nilsson et al., who demonstrated that common ICU scoring systems did not outperform age and GCS in predicting mid-term mortality in ICH patients [16]. In this context, CNS-LAND may be considered a simpler and potentially more practical alternative to APACHE II.

Calibration analysis confirmed that CNS-LAND demonstrated adequate goodness-of-fit across deciles of predicted probability (Hosmer-Lemeshow χ^2^ = 10.931; *p* = 0.206), comparable to GCS (*p* = 0.173), SOFA (*p* = 0.339), and APACHE II (*p* = 0.153). However, the Brier score for CNS-LAND (0.144) was notably higher than those of GCS (0.096) and SOFA (0.107), reflecting its comparatively lower composite predictive accuracy—a finding consistent with its inferior AUC. While all four scores were adequately calibrated, these results reinforce that calibration alone does not distinguish CNS-LAND from its comparators; it is the discrimination gap that limits its clinical utility in this population.

Decision curve analysis provided further insight into the comparative clinical utility of these scores. CNS-LAND offered meaningful net benefit primarily at threshold probabilities ≤25%, consistent with a rule-out role at lower risk thresholds. At threshold probabilities ≥30%—which are more clinically relevant for decisions to escalate monitoring or intervention—its net benefit diminished markedly and approached zero at 50%, at which point GCS continued to demonstrate substantial net benefit. This divergence provides additional evidence that GCS offers more robust and consistent decision-making utility across the full spectrum of clinically relevant threshold probabilities in ICU patients with spontaneous ICH.

Several limitations of this study should be acknowledged. First, the retrospective, single-center design introduces the potential for selection bias, and the findings cannot be generalized without prospective evaluation in external cohorts. Second, key radiological variables with well-established, independent prognostic weight in ICH—including hematoma volume, hematoma location, presence of intraventricular hemorrhage, hydrocephalus, and midline shift—were not systematically extracted from imaging reports and could not be incorporated into the analysis. Because these variables are precisely the determinants that structure the ICH Score, and none of them is captured by CNS-LAND, GCS, SOFA, or APACHE II, their omission is a substantive limitation rather than an incidental one [4]. It prevented calculation of the ICH Score as a direct comparator, and it leaves open the possibility that CNS-LAND’s residual, GCS-independent association with mortality—however small in this cohort—partly reflects unmeasured radiological severity rather than the biochemical processes the score was designed to capture. Furthermore, the cohort comprised exclusively supratentorial ICH cases, which limits the generalizability of the findings to infratentorial hemorrhages such as cerebellar and brainstem bleeding. Third, biomarkers were measured only at admission, and their temporal dynamics were not assessed. Fourth, because CNS-LAND was originally developed to predict early neurological deterioration, its evaluation against a different outcome measure—ICU mortality—in the present study inevitably introduces a degree of outcome mismatch. Finally, patients who died within the first 24 h of ICU admission were excluded because complete laboratory and clinical data required for score calculation were not available within this timeframe. Although only one patient was excluded on this basis, this subgroup likely represents the most critically ill cases, and their exclusion may have led to an overestimation of the predictive performance of the evaluated scoring systems. Another point that warrants consideration is the use of ICU mortality as the primary outcome. In the Turkish healthcare system, intensive care services are organized according to the level of organ support provided (Level 1–3), rather than by a distinction between acute and chronic care. For patients with severe neurological injury who survive the acute phase but require prolonged life support, such as tracheostomy or home mechanical ventilation, all preparations for transfer to long-term or palliative care facilities are generally completed within the ICU. Consequently, ICU mortality is expected to closely approximate hospital mortality in our setting, minimizing the potential for bias arising from inter-facility transfers or early discharge. Nevertheless, this organizational structure may differ across healthcare systems, and this should be taken into account when interpreting and generalizing our findings to other settings.

## 5. Conclusions

This study demonstrates that the CNS-LAND score is significantly associated with ICU mortality in patients with spontaneous ICH, with discriminative performance comparable to APACHE II and adequate calibration across risk categories. However, multivariable analyses showed that this predictive power is largely attributable to the score’s NIHSS component, which carries highly overlapping information with the Glasgow Coma Scale in patients with space-occupying ICH. While this indicates that CNS-LAND does not provide an independent prognostic contribution beyond GCS, its high negative predictive value does not, on its own, establish an independent clinical role, given its strong dependence on GCS, and should not be regarded as a replacement for standard neurological assessment or established ICU severity scoring systems. To the best of our knowledge, this is the first study to systematically evaluate CNS-LAND in spontaneous ICH; further studies are needed to determine whether an ICH-specific version could offer incremental prognostic value.

## Figures and Tables

**Table 1:** Baseline characteristics and between-group comparison of survivors and non-survivors among ICU patients with non-traumatic spontaneous intracerebral hemorrhage (*n* = 305).

Variable	Total (*n* = 305)	Survivors (*n* = 234)	Non-Survivors (*n* = 71)	*p* Value
Demographics				
Age, years, median (IQR)	66.0 (55.0–77.0)	65.5 (54.0–75.0)	68.0 (58.0–80.0)	0.067
Male sex, *n* (%)	191 (62.6)	147 (62.8)	44 (62.0)	0.897
Comorbidities				0.007
None	53 (17.4)	44 (18.8)	9 (12.7)	
Single	91 (29.8)	78 (33.3)	13 (18.3)	
Multiple	161 (52.8)	112 (47.9)	49 (69.0)	
Clinical and ICU Data				
GCS, median (IQR)	12.0 (6.0–15.0)	14.0 (9.0–15.0)	4.0 (4.0–6.0)	<0.001
NIHSS, median (IQR)	10.0 (4.0–26.0)	5.0 (3.0–16.2)	38.0 (26.0–40.0)	<0.001
Systolic BP, mmHg	145.0 (130.0–160.0)	150.0 (135.0–165.0)	120.0 (100.0–145.0)	<0.001
ICU length of stay, days	10.0 (5.0–20.0)	10.0 (5.0–20.0)	13.0 (4.0–28.0)	0.274
Mechanical ventilation, *n* (%)	120 (39.3)	49 (20.9)	71 (100.0)	<0.001
Prognostic Scores				
APACHE II	12.0 (7.0–17.0)	10.0 (5.75–15.0)	18.0 (16.0–24.0)	<0.001
SOFA	4.0 (2.0–6.0)	3.0 (1.0–5.0)	7.0 (6.0–9.0)	<0.001
CNS-LAND	4.0 (2.0–5.0)	3.0 (2.0–5.0)	6.0 (5.0–6.0)	<0.001
Hematology				
WBC, ×10^9^/L	10.5 (7.9–14.4)	10.2 (7.7–13.3)	12.7 (8.7–16.7)	0.003
Neutrophil, %	79.7 (63.9–87.5)	79.4 (63.6–87.3)	81.1 (66.6–87.3)	0.536
Platelet, ×10^9^/L	231 (183.5–293.5)	237 (187–297)	227 (179–275)	0.057
Biochemistry				
BUN, mmol/L	16.2 (12.8–22.7)	16.0 (12.5–21.2)	19.4 (12.9–30.7)	0.016
Creatinine, mg/dL	0.8 (0.6–1.0)	0.8 (0.6–1.0)	0.9 (0.7–1.5)	0.001
AST, U/L	21.0 (17.0–28.0)	20.0 (16.7–27.0)	22.0 (17.0–30.0)	0.064
ALT, U/L	16.0 (12.0–25.0)	16.0 (12.0–24.0)	16.0 (12.0–31.0)	0.340
LDH, U/L	224 (174.5–284.5)	214 (168–260)	296 (222–342)	<0.001
CK-MB, ng/mL	2.3 (1.6–4.0)	2.1 (1.5–3.6)	3.4 (2.0–6.0)	<0.001
CRP, mg/L	10.0 (3.1–36.3)	8.6 (3.0–31.2)	12.5 (5.6–61.1)	0.013
Procalcitonin, ng/mL	0.1 (0.0–0.2)	0.1 (0.0–0.1)	0.2 (0.0–0.5)	<0.001
Coagulation				
PT, s	9.2 (8.7–10.0)	9.1 (8.7–9.8)	9.7 (8.9–10.9)	<0.001
INR	1.0 (0.9–1.0)	1.0 (0.9–1.0)	1.0 (0.9–1.1)	<0.001
APTT, s	27.6 (24.8–31.9)	27.4 (24.7–31.4)	28.5 (25.2–33.7)	0.149
D-dimer, mg/L	1.0 (0.7–2.9)	1.0 (0.7–2.5)	2.0 (0.8–6.0)	<0.001

Data are presented as median (IQR) or number (percentage). Continuous variables were compared using the Mann–Whitney U test; categorical variables were compared using the chi-square test or Fisher’s exact test as appropriate. Complete separation: all non-survivors were receiving mechanical ventilation; Fisher’s exact test could not be applied; *p* < 0.001 is reported. Abbreviations: GCS, Glasgow Coma Scale; NIHSS, National Institutes of Health Stroke Scale; BP, blood pressure; ICU, intensive care unit; APACHE II, Acute Physiology and Chronic Health Evaluation II; SOFA, Sequential Organ Failure Assessment; WBC, white blood cell; BUN, blood urea nitrogen; AST, aspartate aminotransferase; ALT, alanine aminotransferase; LDH, lactate dehydrogenase; CK-MB, creatine kinase MB isoenzyme; CRP, C-reactive protein; PT, prothrombin time; INR, international normalized ratio; APTT, activated partial thromboplastin time; IQR, interquartile range.

**Table 2:** Discriminative performance of prognostic scores for ICU mortality in ICU patients with non-traumatic spontaneous intracerebral hemorrhage.

Score	AUC (95% CI)	Optimal Cut-Off	Sens (%)	Spec (%)	PLR	NLR	PPV (%)	NPV (%)	*p* Value
GCS	0.925 (0.893–0.950)	≤11	100.0	69.7	3.30	0.00	50.0	100.0	<0.001
SOFA	0.906 (0.871–0.937)	≥6	85.9	81.6	4.68	0.173	58.7	95.0	<0.001
APACHE II	0.840 (0.786–0.887)	≥15	84.5	71.8	3.00	0.216	47.6	93.9	0.467
CNS-LAND	0.816 (0.767–0.861)	≥5	83.1	70.9	2.86	0.238	46.5	93.3	—

Abbreviations: AUC, area under the receiver operating characteristic curve; CI, confidence interval; Sens, sensitivity; Spec, specificity; PLR, positive likelihood ratio; NLR, negative likelihood ratio; PPV, positive predictive value; NPV, negative predictive value; GCS, Glasgow Coma Scale; SOFA, Sequential Organ Failure Assessment; APACHE II, Acute Physiology and Chronic Health Evaluation II. Optimal cut-off values were determined by the Youden J index. 95% CIs were derived from 2000-iteration bootstrap resampling. PLR and NLR are prevalence-independent measures of diagnostic accuracy and are reported because PPV and NPV are influenced by the observed ICU mortality rate in this cohort (23.3%). *p* values represent pairwise AUC comparisons against CNS-LAND assessed by bootstrap-based DeLong test; — denotes the reference score.

**Table 3:** Pairwise AUC comparisons between CNS-LAND and ICU severity scores by bootstrap-based DeLong method.

Comparison	ΔAUC (95% CI)	*p* Value	Interpretation
CNS-LAND vs. GCS	−0.109 (−0.151 to −0.067)	<0.001	GCS superior
CNS-LAND vs. SOFA	−0.090 (−0.138 to −0.040)	<0.001	SOFA superior
CNS-LAND vs. APACHE II	−0.024 (−0.088 to +0.040)	0.467	No significant difference

Abbreviations: ΔAUC, difference in area under the curve (CNS-LAND minus comparator); a negative ΔAUC indicates the comparator score has a higher AUC. CI, confidence interval; GCS, Glasgow Coma Scale; SOFA, Sequential Organ Failure Assessment; APACHE II, Acute Physiology and Chronic Health Evaluation II. Bootstrap resampling: 2000 iterations.

**Table 4:** Spearman correlations, partial correlation analysis, and logistic regression model comparisons.

Analysis/Variable	Statistic	95% CI	*p* Value
Spearman rank correlation			
CNS-LAND~GCS	rs = −0.772	—	<0.001
CNS-LAND~SOFA	rs = +0.666	—	<0.001
CNS-LAND~APACHE II	rs = +0.577	—	<0.001
NIHSS~GCS †	rs = −0.964	—	<0.001
Partial Spearman correlation (controlling for GCS)			
CNS-LAND~Mortality (unadjusted)	rs = +0.468	—	<0.001
CNS-LAND~Mortality|GCS	rs = −0.051	—	0.373
Logistic regression—primary analysis (GCS as reference)			
Model 1: GCS alone	AUC = 0.925, −2LL = 179.430	0.902–0.954	—
Model 2: GCS + CNS-LAND	AUC = 0.925, −2LL = 179.310	0.903–0.955	—
LRT (Model 2 vs. Model 1)	χ^2^ = 0.121, df = 1	—	0.728
CNS-LAND OR (Model 2)	OR = 0.949	0.709–1.272	0.728
ΔAUC (Model 2 − Model 1)	0.000	−0.002 to +0.004	0.590
IDI	0.001	—	—
Logistic regression—broader multivariable analysis (GCS + age + comorbidity)			
Model A: GCS + age + comorbidity	AUC = 0.928, −2LL = 176.269	—	—
GCS	OR = 0.583	0.507–0.669	<0.001
Age	OR = 1.024	0.992–1.057	0.147
Comorbidity	OR = 1.048	0.576–1.905	0.878
Model B: Model A + CNS-LAND	AUC = 0.927, −2LL = 176.108	—	—
LRT (Model B vs. Model A)	χ^2^ = 0.161, df = 1	—	0.688
CNS-LAND OR (Model B)	OR = 0.941	0.701–1.265	0.688

rs, Spearman rank correlation coefficient; OR, odds ratio; CI, confidence interval; LRT, likelihood ratio test; IDI, Integrated Discrimination Improvement; −2LL, −2 log likelihood; GCS, Glasgow Coma Scale; SOFA, Sequential Organ Failure Assessment; APACHE II, Acute Physiology and Chronic Health Evaluation II; NIHSS, National Institutes of Health Stroke Scale. AUC 95% CIs derived from 2000-iteration bootstrap resampling. † NIHSS~GCS collinearity is reported to identify the mechanistic basis of CNS-LAND’s redundancy with GCS in ICH patients. Mechanical ventilation was excluded from all regression models due to complete separation (100% of non-survivors required mechanical ventilation), which precludes logistic regression estimation. SOFA and APACHE II were not included as additional covariates because both scores incorporate GCS directly, introducing collinearity with the primary predictor of interest.

**Table 5:** Hosmer-Lemeshow goodness-of-fit and Brier score results for each prognostic score.

Score	AUC	H-L χ^2^	*p* Value	Brier Score	Calibration
GCS	0.925	11.543	0.173	0.096	Good
SOFA	0.906	9.043	0.339	0.107	Good
APACHE II	0.840	11.955	0.153	0.134	Good
CNS-LAND	0.816	10.931	0.206	0.144	Good

H-L, Hosmer-Lemeshow; AUC, area under the receiver operating characteristic curve. Hosmer-Lemeshow test: 10 groups, df = 8; *p* > 0.05 indicates adequate calibration. Brier score: lower values indicate better overall predictive accuracy (discrimination + calibration combined); a null model (event rate only) yields a Brier score of 0.179 in this cohort.

## Data Availability

The data presented in this study are available on request from the corresponding author due to patient privacy and confidentiality restrictions, in accordance with the requirements of the institutional ethics committee.
